# Association Between Bitter Taste Receptor Phenotype and Clinical Outcomes Among Patients With COVID-19

**DOI:** 10.1001/jamanetworkopen.2021.11410

**Published:** 2021-05-25

**Authors:** Henry P. Barham, Mohamed A. Taha, Stephanie T. Broyles, Megan M. Stevenson, Brittany A. Zito, Christian A. Hall

**Affiliations:** 1 Rhinology and Skull Base Research Group, Baton Rouge General Medical Center, Baton Rouge, Louisiana; 2 Sinus and Nasal Specialists of Louisiana, Baton Rouge; 3 Department of Otorhinolaryngology, Cairo University, Cairo, Egypt; 4 Pennington Biomedical Research Center, Baton Rouge, Louisiana

## Abstract

**Importance:**

Bitter taste receptors (T2Rs) have been implicated in sinonasal innate immunity, and genetic variation conferred by allelic variants in T2R genes is associated with variation in upper respiratory tract pathogen susceptibility, symptoms, and outcomes. Bitter taste receptor phenotype appears to be associated with the clinical course and symptom duration of SARS-CoV-2 infection.

**Objective:**

To evaluate the association between T2R phenotype and patient clinical course after infection with SARS-CoV-2.

**Design, Setting, and Participants:**

A prospective cohort study was performed from July 1 through September 30, 2020, at a tertiary outpatient clinical practice and inpatient hospital in the United States among 1935 participants (patients and health care workers) with occupational exposure to SARS-CoV-2.

**Exposure:**

Exposure to SARS-CoV-2.

**Main Outcomes and Measures:**

Participants underwent T2R38 phenotype taste testing to determine whether they were supertasters (those who experienced greater intensity of bitter tastes), tasters, or nontasters (those who experienced low intensity of bitter tastes or no bitter tastes) and underwent evaluation for lack of infection with SARS-CoV-2 via polymerase chain reaction (PCR) testing and IgM and IgG testing. A group of participants was randomly selected for genotype analysis to correlate phenotype. Participants were followed up until confirmation of infection with SARS-CoV-2 via PCR testing. Phenotype of T2R38 was retested after infection with SARS-CoV-2. The results were compared with clinical course.

**Results:**

A total of 1935 individuals (1101 women [56.9%]; mean [SD] age, 45.5 [13.9] years) participated in the study. Results of phenotype taste testing showed that 508 (26.3%) were supertasters, 917 (47.4%) were tasters, and 510 (26.4%) were nontasters. A total of 266 participants (13.7%) had positive PCR test results for SARS-CoV-2. Of these, 55 (20.7%) required hospitalization. Symptom duration among patients with positive results ranged from 0 to 48 days. Nontasters were significantly more likely than tasters and supertasters to test positive for SARS-CoV-2 (odds ratio, 10.1 [95% CI, 5.8-17.8]; *P* < .001), to be hospitalized once infected (odds ratio, 3.9 [1.5-10.2]; *P* = .006), and to be symptomatic for a longer duration (mean [SE] duration, 23.7 [0.5] days vs 13.5 [0.4] days vs 5.0 [0.6] days; *P* < .001). A total of 47 of 55 patients (85.5%) with COVID-19 who required inpatient admission were nontasters. Conversely, 15 of 266 patients (5.6%) with positive PCR test results were supertasters.

**Conclusions and Relevance:**

This cohort study suggests that T2R38 receptor allelic variants were associated with participants’ innate immune response toward SARS-CoV-2. The T2R phenotype was associated with patients’ clinical course after SARS-CoV-2 infection. Nontasters were more likely to be infected with SARS-CoV-2 than the other 2 groups, suggesting enhanced innate immune protection against SARS-CoV-2.

## Key Points

**Question:**

What is the association between the bitter taste receptor phenotype and outcomes after infection with SARS-CoV-2?

**Findings:**

In this cohort study of 1935 adults, 266 tested positive for SARS-CoV-2, and those who experienced low intensity of bitter tastes or no bitter tastes (nontasters) were significantly more likely to test positive for SARS-CoV-2, to be hospitalized, and to be symptomatic for a longer duration. Conversely, those who experienced greater intensity of bitter tastes (supertasters) represented 5.6% of patients infected with SARS-CoV-2, suggesting enhanced innate immune protection.

**Meaning:**

This study suggests that bitter taste receptor allelic variants are associated with innate immune fitness toward SARS-CoV-2 and can be used to correlate with clinical course and prognosis of COVID-19.

## Teaser

This cohort study evaluates the association between bitter taste receptor phenotype and patient clinical course after infection with SARS-CoV-2.

## Introduction

A cluster of viral pneumonia cases associated with a novel coronavirus (2019-nCoV) first identified in Wuhan, Hubei Province, China, in December 2019 has rapidly spread around the world, causing a global health crisis. The disease was subsequently named coronavirus disease 2019 (COVID-19) by the World Health Organization and has been designated severe acute respiratory syndrome coronavirus 2 (SARS-CoV-2). Significant concern has arisen within the global community regarding the potential risks of infectious transmission of SARS-CoV-2.^1^

Factors such as social and psychological stress, economic hardship, and inconsistent virulence of SARS-CoV-2 are likely associated with the apparent lack of adherence to the advised behavior modifications. The ability to identify individuals whose health is most at risk by SARS-CoV-2 may allow society to balance social reengagement more efficiently with protection of public health. School attendance, mass gatherings, travel, and other such public activities may be able to more fully resume while we await the development of novel therapeutics.

Given human immunologic naivete to SARS-CoV-2, the innate immune system may play an important role in the defense against the virus. A growing body of literature has suggested a role for bitter taste receptors (T2Rs) in sinonasal innate immunity^2,3,4,5^; these extraoral T2Rs are present on ciliated epithelial cells and solitary chemosensory cells. T2R38, one of the many isoforms of T2Rs, is a receptor that is localized to motile cilia in humans, agonized by phenylthiocarbamide (PTC) and propylthiouracil (PROP).^6^ When T2R38 is stimulated by agonists, nitric oxide (NO) is produced to increase mucociliary clearance and kill pathogens in the human respiratory tract mucosa.^3^

In a prior study evaluating the association of NO with SARS-CoV, Åkerström et al^7^ found that NO inhibits the replication of SARS-CoV by 2 distinct mechanisms. First, NO or its derivatives may cause a reduction in the palmitoylation of a nascently expressed spike protein, which affects the fusion between the spike protein and its cognate receptor, angiotensin-converting enzyme 2. Second, NO or its derivatives may cause a reduction in viral RNA production in the early steps of viral replication, which may possibly be due to an effect on 1 or both of the cysteine proteases encoded in Orf1 of SARS-CoV.

Three single-nucleotide variations in the gene that encodes T2R38, *TAS2R38* (GenBank AY258597), confer 2 common haplotypes, including the functional variant *PAV* (proline-alanine-valine) and the nonfunctional variant *AVI* (alanine-valine-isoleucine). Homozygotes for the functional allele (*PAV/PAV*) perceive T2R38 agonists, such as PTC and PROP, as intensely bitter, whereas homozygotes for the nonfunctional allele (*AVI/AVI*) are unable to perceive these compounds. Heterozygotes (*PAV/AVI*) demonstrate a wide range of bitter taste perceptions depending on the level of expression of the nonfunctional and functional alleles.^8,9^ Sinonasal epithelial cells cultured from individuals with *AVI/AVI* compared with cells cultured from individuals with *PAV/PAV* also demonstrate reduced NO release with a resultant decrease in ciliary beat frequency and mucociliary clearance. Compared with patients with chronic rhinosinusitis and *PAV/PAV*, those with *AVI/AVI* also demonstrate increased susceptibility to upper respiratory tract infections^10,11^ (eFigure 1 in the Supplement).

Prior studies have shown evidence for an association between the PTC or PROP taste test and sinonasal innate immunity, concluding that the ability to assess airway taste receptor variation with an inexpensive taste test has broad implications because differences in airway taste receptor function may reflect impaired innate immunity and a predisposition to certain respiratory tract infections and inflammatory disorders, and T2R38 functionality in the tongue correlates with nasal symptoms in healthy individuals.^12,13^

In a retrospective study performed by Barham et al^1^ on 100 positive cases of COVID-19 confirmed by polymerase chain reaction (PCR), phenotypic expression of T2R38 with taste strip testing appeared to be associated with the clinical course and symptoms specific to each individual because 100% of the patients requiring inpatient admission were classified as nontasters (those who experienced low intensity of bitter tastes or no bitter tastes). Conversely, supertasters (those who experienced greater intensity of bitter tastes) represented 0% of the patient population, suggesting the possibility of innate immunity to SARS-CoV-2.

We set out to identify an association between T2R genotype with phenotype and outcomes after infection with COVID-19. We present our findings as an area that warrants further scientific study to potentially create a safe, cost-effective, accurate, and easily scalable screening tool that has the potential to stratify patients into groups and assess the risk of infection with SARS-CoV-2 and the expected clinical course of the disease.

## Methods

We performed a prospective cohort study at our outpatient clinical practice and inpatient hospital from July 1 through September 30, 2020, of patients and health care workers with exposure to SARS-CoV-2. Participants underwent phenotype taste testing and an evaluation for lack of infection with SARS-CoV-2 via PCR testing (to exclude current infection) and IgM and IgG testing (to exclude previous infection). All participants were categorized into 3 groups (supertasters, tasters, and nontasters) via phenotypic expression of T2R38. A group of participants was randomly selected for genotype analysis at the Monell Chemical Senses Center, Philadelphia, Pennsylvania, using Oragene collection kits (DNA Genotek) to correlate phenotype. Participants were followed up until infection with SARS-CoV-2 was confirmed via PCR test results. Data on phenotype expression of T2R38 were again collected after infection, and the results of both genotype and phenotype were compared with clinical course and outcome of disease. We stratified patients into more-severe and less-severe clinical courses of disease according to the need for hospitalization during the infected period; patients requiring hospitalization for treatment compose the cohort with a more severe form of infection, and those not requiring hospitalization compose the cohort with the less severe form of infection (eFigure 2 in the Supplement). Written informed consent was obtained from study participants. All aspects of this study were reviewed and approved by the Baton Rouge General institutional review board. This study followed the Strengthening the Reporting of Observational Studies in Epidemiology (STROBE) reporting guideline.

Phenotype expression of T2R38 was evaluated via taste strip tests to assess the genetically determined taste response phenotype of each participant. This study used an early prototype general wellness test kit, which is being developed along with a software function and is now owned by Phenomune LLC. This test is designed to be used by persons at home to detect, interpret, record, and produce a trait report describing their unique intensity level of phenotypic expression of T2Rs intended to increase their awareness to sensitivity to bitter tastes for general improvement of functions associated with a general state of health, such as healthy lifestyle choices to enable wellness monitoring as it relates to dietary choices. There are several other commercially available taste strip tests (eg, those sold by Bartovation LLC and Eisco Labs); however, an earlier prototype test kit consistent with the Phenomune general wellness test kit was used by the investigators in this study owing to its proprietary interpretation system for determining the scaled intensity of expression to facilitate a more precise classification of each participant. These taste strip tests included a control (chemical free), PTC, thiourea, and sodium benzoate.

### Demonstration and Interpretation of the Taste Strip Test

All participants were presented with the taste test strips in the following order: (1) control strip, (2) PTC strip, (3) thiourea strip, and (4) sodium benzoate strip. Participants were instructed to place the provided litmus paper strip on their tongue until completely moistened, then the next litmus paper strip was provided according to the order already stated. Participants were instructed to comment on the quality of taste they perceived, in addition to commenting on its intensity on a visual analog scale from 0 to 10, where 0 is no perception and 10 is extremely intense quality perceived compared with the control strip. Each participant was oriented to the scale with a verbal explanation prior to proceeding. In between each taste strip provided, participants were allowed to sip water.

Subsequent SARS-CoV-2 infection was confirmed via PCR, and participants’ taste phenotypes were reassessed at diagnosis. Outcomes associated with severity of disease were assessed via medical records.

### Exclusions

Participants with evidence of active infection with SARS-CoV-2 confirmed via positive PCR test result at study commencement were excluded. Participants with evidence of prior infection with SARS-CoV-2 via a positive IgM and/or IgG result at study commencement were excluded. Participants were excluded from evaluation if they had positive results on the control strip.

### Statistical Analysis

Statistical analyses were performed using SAS, version 9.4 (SAS Institute Inc). Unadjusted comparisons of baseline characteristics and outcomes were conducted via χ^2^ tests and analyses of variance. Logistic regression analyses and zero-inflated Poisson analysis were used to assess associations between tasting phenotypes and clinical course; all models were adjusted for age and sex. All *P* values were from 1-sided tests, and results were deemed statistically significant at *P* < .05.

## Results

A total of 1935 individuals (1101 women [56.9%]; mean [SD] age, 45.5 [13.9] years) were assessed by phenotype taste testing. All participants were categorized into 3 groups (supertasters, tasters, and nontasters) via phenotypic expression of T2R38: 508 (26.3%) were categorized as supertasters, 917 (47.4%) were categorized as tasters, and 510 (26.4%) were categorized as nontasters (Table 1).

**Table 1.** Classification of Participants

Characteristic	Participants, No. (%)				*P* value
	Overall	Nontaster	Taster	Supertaster	
No. (%)	1935 (100)	510 (26.4)	917 (47.4)	508 (26.3)	NA
Baseline characteristics					
Age, mean (SD), y	45.5 (13.9)	49.1 (15.9)	45.6 (13.4)	41.6 (11.2)	<.001
Sex					
Female	1101 (56.9)	290 (56.9)	467 (50.9)	344 (67.7)	<.001
Male	834 (43.1)	220 (43.1)	450 (49.1)	164 (32.3)	
Outcomes					
Positive SARS-CoV-2 test result	266 (13.7)	147/266 (55.3)	104/266 (39.1)	15/266 (5.6)	<.001
Hospitalization^a^	55 (20.7)	47/55 (85.5)	8/55 (14.5)	0/55	<.001
Symptom duration, mean (SD), d^a^	18.7 (7.7)	23.7 (5.2)	13.5 (4.8)	5.0 (2.0)	<.001

Abbreviation: NA, not applicable.

^a^ Among those with a positive SARS-CoV-2 test result.

**Figure 1.**
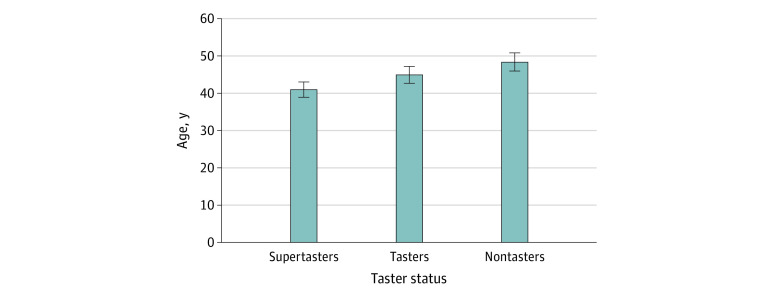
Age Distribution Among Taster Groups Error bars indicate mean (SD) age in years.

When evaluating the 3 groups at baseline, our results show decreasing phenotypic expression with increasing age. The mean (SD) age of the supertaster group was 41.6 (11.2) years, the mean (SD) age of the taster group was 45.6 (13.4) years, and the mean (SD) age of the nontaster group was 49.1 (15.9) years (*P* < .001) (Table 1 and Figure 1).

A total of 266 participants (13.7%) were considered to have positive PCR results for SARS-CoV-2. Only 15 of these participants (5.6%) were categorized as supertasters prior to infection; 104 (39.1%) were categorized as tasters prior to infection, and 147 (55.3%) were categorized as nontasters prior to infection (*P* < .001).

Among the 266 participants with COVID-19, the most common symptoms were fever, nasal congestion, cough, shortness of breath, loss of smell, and headache (Table 2). The most common comorbidities were diabetes, hypertension, rhinosinusitis, asthma, and cardiac disease.

**Table 2.** Clinical Features and Comorbidities of Patients With Positive SARS-CoV-2 Test Result

Clinical feature	Patients, No. (%) (n = 266)
Symptoms	
Fever (>38.0° C)	208 (78.2)
Nasal congestion	165 (62.0)
Cough	150 (56.4)
Shortness of breath	139 (52.3)
Loss of smell	135 (50.8)
Headache	106 (39.8)
Myalgia	45 (16.9)
Gastrointestinal	9 (3.4)
Comorbidities	
Diabetes	48 (18.0)
Hypertension	40 (15.0)
Rhinosinusitis (chronic and recurrent acute)	35 (13.2)
Asthma	21 (7.9)
Cardiac disease	18 (6.8)
Autoimmune disease	13 (4.9)
Carcinoma	8 (3.0)
Chronic renal failure	5 (1.9)

Patients were stratified into more-severe and less-severe clinical courses of disease according to the need for hospitalization during the infected period. Fifty-five of the participants with COVID-19 (20.7%) required hospitalization for severity of disease. None of the hospitalized patients were categorized as supertasters prior to infection, 8 (14.5%) were categorized as tasters prior to infection, and 47 (85.5%) were categorized as nontasters prior to infection (*P* < .001) (Table 1). The mean (SD) age of tasters requiring admission was 74.0 (3.9) years.

**Figure 2.**
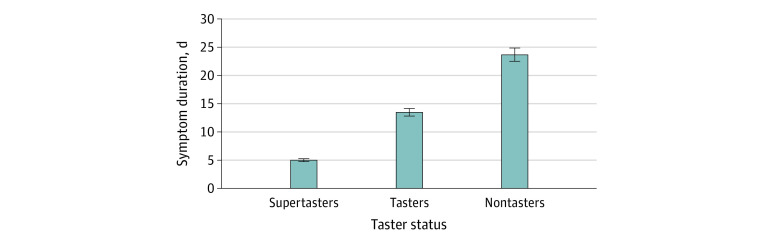
Symptom Duration Among Taster Groups of Patients Positive for SARS-CoV-2 Error bars indicate mean (SD) duration in days.

We evaluated symptom duration for patients with SARS CoV-2 confirmed via positive PCR results and found that the mean duration ranged from 0 to 48 days. The mean (SD) symptom duration was 5.0 (2.0) days for supertasters, 13.5 (4.8) days for tasters, and 23.7 (5.2) days for nontasters (*P* < .001) (Table 1 and Figure 2).

Nontasters were significantly more likely than tasters and supertasters to test positive for SARS-CoV-2 (odds ratio, 10.1 [95% CI, 5.8-17.8]; *P* < .001), to be hospitalized once infected (odds ratio, 3.9 [95% CI, 1.5-10.2]; *P* = .006), and to be symptomatic for a longer duration (mean [SE], 23.7 [0.5] days vs 13.5 [0.4] days vs 5.0 [0.6] days; *P* < .001) (Table 3). The risk of infection and of symptom duration showed significant evidence of linear trends across the tasting phenotypes. When evaluating the association between phenotype and genotype, phenotype showed 94.2% (49 of 52) accuracy in assessing genotype.

**Table 3.** Associations Between Taster Status, SARS-CoV-2 Infection, and Clinical Consequences of Participants With Positive SARS-CoV-2 Test Results

Taster status	Positive SARS-CoV-2 test result, %		Hospitalized, %		Symptom duration, mean (SE), d^a^
	Mean (SE)^a^	OR (95% CI)	Mean (SE)^a^	OR (95% CI)	
Nontaster	25.8 (2.0)	10.1 (5.8-17.8)	12.9 (3.5)	3.9 (1.5-10.2)	23.7 (0.5)
Taster	10.5 (1.0)	3.4 (1.9-6.0)	3.7 (1.8)^b^	1 [Reference]^b^	13.5 (0.4)
Supertaster	3.3 (0.8)	1 [Reference]			5.0 (0.6)
*P* value	<.001^c^	NA	.006	NA	<.001^c^

Abbreviations: NA, not applicable; OR, odds ratio.

^a^ Least-squares mean estimates from models adjusting for age and sex.

^b^ Tasters and supertasters pooled for analysis because there were no hospitalizations among supertasters.

^c^ Test for linear trend (dose-response association) across taster phenotypes.

We evaluated phenotypic expression with taste perception for both quality and intensity prior to and during infection with SARS-CoV-2 in the cohort of 266 patients with positive PCR test results for SARS-CoV-2. No patient reported a change in the quality of taste perceived during infection. There were no changes in the grouping of patients. In addition, no patient reported a change in intensity greater than 2 points on our visual analog taste intensity scale (associated with subjective differences).

## Discussion

A prior study of SARS-CoV-2 has demonstrated that the spike protein binds to the angiotensin converting enzyme 2 with enhancement by proteolytic cleavage of the spike protein by serine protease, which is distributed in the respiratory tract epithelium, the lung parenchyma, and other areas (such as the gastrointestinal tract and endothelial cells).^14,15^ Åkerström et al^7^ have shown that NO can inhibit the fusion of the spike protein to angiotensin converting enzyme 2 and that NO may also inhibit early production of viral RNA. Activation of extraoral T2Rs has been shown to promote release of NO from epithelial ciliated cells,^2^ which increases the ciliary beat frequency of these cells to clear invading pathogens. The data in our study suggest that the *T2R38* genotype correlates with the T2R38 phenotype. The data also suggest that the T2R38 phenotype is associated with the clinical course in individuals infected with SARS-CoV-2. The T2R38 phenotype may help clinicians assess patients’ innate immune fitness toward SARS-CoV-2. In the setting of a novel virus, the adaptive immune system cannot play an immediate role in host defense. Innate immune protection against a viral pathogen becomes paramount when an efficient adaptive immune response cannot be mounted. Our data suggest that the T2Rs may play a vital role in protection against SARS-CoV-2 by enhancing the host’s innate immune response against SARS-CoV-2.

In the extraoral airway, T2Rs do not modulate taste sensation. In this setting, these receptors are present on a variety of cell types, with T2Rs present on epithelial ciliated cells, which play a role in innate immune defense when they bind to their specific agonists. Bitter taste perception is mediated by a family of approximately 25 T2Rs called the taste receptor family 2, and these T2Rs respond to a variety of bitter compounds, such as PTC, denatonium benzoate, strychnine, quinine, and caffeine.^16,17^

Ciliated sinonasal epithelial cells are an essential component of the first line of defense in upper airway tract immunity. Effective mucociliary clearance requires the coordinated ciliary-driven movement of airway surface liquid, composed of mucus-trapped pathogenic organisms and debris, to maintain a healthy sinonasal tract. When mucociliary clearance is impaired, stasis of sinonasal secretions and resultant local inflammation occur and can be an inciting factor in increasing susceptibility to infection.^18,19,20,21,22^

These immunoprotective mechanisms are triggered by the recognition of microbial pathogens, which occurs via activation of several receptor types. It was found that T2Rs were able to recognize pathogens and elicit downstream responses within a matter of minutes, unlike the toll-like receptors that recognize the conserved pathogen-associated molecular patterns and take as long as 12 hours to elicit a downstream gradual immune response owing to changes in gene expression. The mechanism by which this response occurs in the sinonasal epithelium has been a topic of investigation for the past decade.^23^

The innate immune responses elicited via activation of T2R38 include Ca^2+^-driven NO production. This calcium and NO signaling involves 2 canonical components of the classic taste signaling cascade first described in type 2 taste cells, namely, an isoform of phospholipase C (PLCβ2)^24,25^ and the TRPM5 ion channel.^26^ Nitric oxide induces damage to the intracellular components of infectious microbes and, via its action on protein kinase G and guanylyl cyclase, increases the ciliary beat frequency, thereby increasing mucociliary clearance.^4,19,27,28^ This increase in the ciliary beat frequency accelerates the removal of mucus-trapped pathogens and the dispersion of other antimicrobial compounds produced in response to pathogens.^29,30^

Taste receptors in the upper airway are not limited to ciliated epithelial cells. Solitary chemosensory cells are rare, nonciliated, epithelial cells that express both sweet (T1R2/3) and bitter (T2R) receptors. Although T2R stimulation on ciliated epithelial cells elicits a Ca^2+^-dependent NO response, stimulation of solitary chemosensory cell T2Rs results in the propagation of Ca^2+^ across gap junctions into ciliated cells, triggering them to release antimicrobial compounds, including β defensins 1 and 2, lactoferrin, and others.^31,32^

Beyond T2R38, studies have investigated other T2Rs, with similar findings of ubiquitous expression in the human ciliated sinonasal epithelium and a bitter ligand–dependent, Ca^2+^-mediated NO production.^33,34^ Recent research has explored the use of oral taste sensitivity as a proxy for extraoral taste receptor function and has hypothesized that receptor sensitivity as assessed with a simple taste test can reveal differences in sinonasal mucosal immunity.^12,13^

Multiple studies have evaluated the phenotypic perception of PROP bitter sensitivity occurring over a person’s life span and have shown that sensitivity to the T2R38 agonist phenylthiouracil decreases with age.^35,36,37,38^ This finding appears to occur more frequently in *PAV*/*AVI* heterozygotes. Davies et al,^39^ using an age-structured mathematical model of data from 6 countries, estimated that susceptibility to COVID-19 infection in individuals younger than 20 years is approximately half that of adults older than 20 years, with symptoms manifesting in 21% of those aged 10 to 19 years, which increases to 69% in those older than 70 years. These findings are especially of interest in conjunction with the known decrease in T2R phenotypic expression with increasing age.

Given the current state of the global pandemic of COVID-19, we sought a simple test to associate patient outcomes with phenotypic expression of T2R38. Given that differences in taste sensitivities appear to be associated with corresponding functional differences in upper airway immune response, we postulated that receptor sensitivity could be associated with the expected clinical course of patients exposed to SARS-CoV-2. Supertasters account for approximately 25% of the adult population and appear to make up most of the cohort of asymptomatic or noninfected carriers because their innate immune system, including sinonasal mucosal immunity, helps to prevent the systemic infection of the upper respiratory tract by pathogens. Conversely, nontasters account for approximately 25% of the adult population and appear to account for the cohort of patients with intense symptoms and poor clinical course. Tasters account for approximately 50% of the population and appear to demonstrate a wide range of symptoms but generally can mount an appropriate immune response and recover from COVID-19 infection. Based on the data and the results presented in this study, this could draw a path for future research regarding other possible roles of the T2Rs in innate immunity, especially in the form of potential therapeutics, prioritization of vaccinations, and possible roles against other upper respiratory tract pathogens (ie, influenza).

### Limitations

Our study has some limitations, including the small size for genetic analysis of the T2R38, yet we opted to include it because it provided us with insight regarding the phenotype-genotype correlation. Despite the overall large sample size of our study, it can be considered small amid the current pandemic. Despite this, it can be used as an initiative for future studies on T2Rs and SARS-CoV-2. The absence of a control group (another upper respiratory tract viral infection) in our study is another possible limitation; however, given the novelty of the current pandemic, we opted to focus on the T2Rs’ phenotypic expression in association with COVID-19. Our largest limitation in this observational study is the potential for confounding factors and the fact that SARS-CoV-2 is a novel virus, thus preventing prior knowledge of the degree of inoculation, symptoms, and outcomes in different populations.

## Conclusions

The novelty of SARS-CoV-2 has created the current global pandemic and greatly disrupted life throughout the world. Bitter taste receptors appear to play a crucial role in the innate immunity against upper respiratory tract pathogens, and the allelic variants of these receptors define the magnitude of such innate immunity. In this study, phenotypic expression of T2R38 with taste strip testing was associated with the clinical course and symptoms. Further study evaluating the potential for phenotypic expression of T2R38 as a factor associated with disease on a larger scale is warranted. This finding carries potential global implications for our understanding of SARS-CoV-2, in addition to yearly infections with additional viruses, including influenza.

## Notes

Supplement. **eFigure 1.** Schematic Diagram Showing How Classification Into the Different Taste Groups Was Conducted **eFigure 2.** Flowchart Depicting Study Design
