# Supplementary material for: Association Between Bitter Taste Receptor Phenotype and Clinical Outcomes Among Patients With COVID-19
Source: JAMA Netw Open. 2021 May 25;4(5):e2111410. doi: 10.1001/jamanetworkopen.2021.11410 (PMC8150696; doi:10.1001/jamanetworkopen.2021.11410)
Supplement: Supplement. — eFigure 1. Schematic Diagram Showing How Classification Into the Different Taste Groups Was Conducted eFigure 2. Flowchart Depicting Study Design [file jamanetwopen-e2111410-s001.pdf]

Supplementary Online Content

Barham HP, Taha MA, Broyles ST, Stevenson MM, Zito BA, Hall CA. Association between bitter taste receptor phenotype and clinical outcomes among patients with COVID-19. *JAMA Netw Open.* 2021;4(5):e2111410. doi:10.1001/jamanetworkopen.2021.11410

eFigure 1. Schematic Diagram Showing How Classification Into the Different Taste Groups Was Conducted

eFigure 2. Flowchart Depicting Study Design

This supplementary material has been provided by the authors to give readers additional information about their work.

© 2021 Barham HP et al. *JAMA Network Open.*

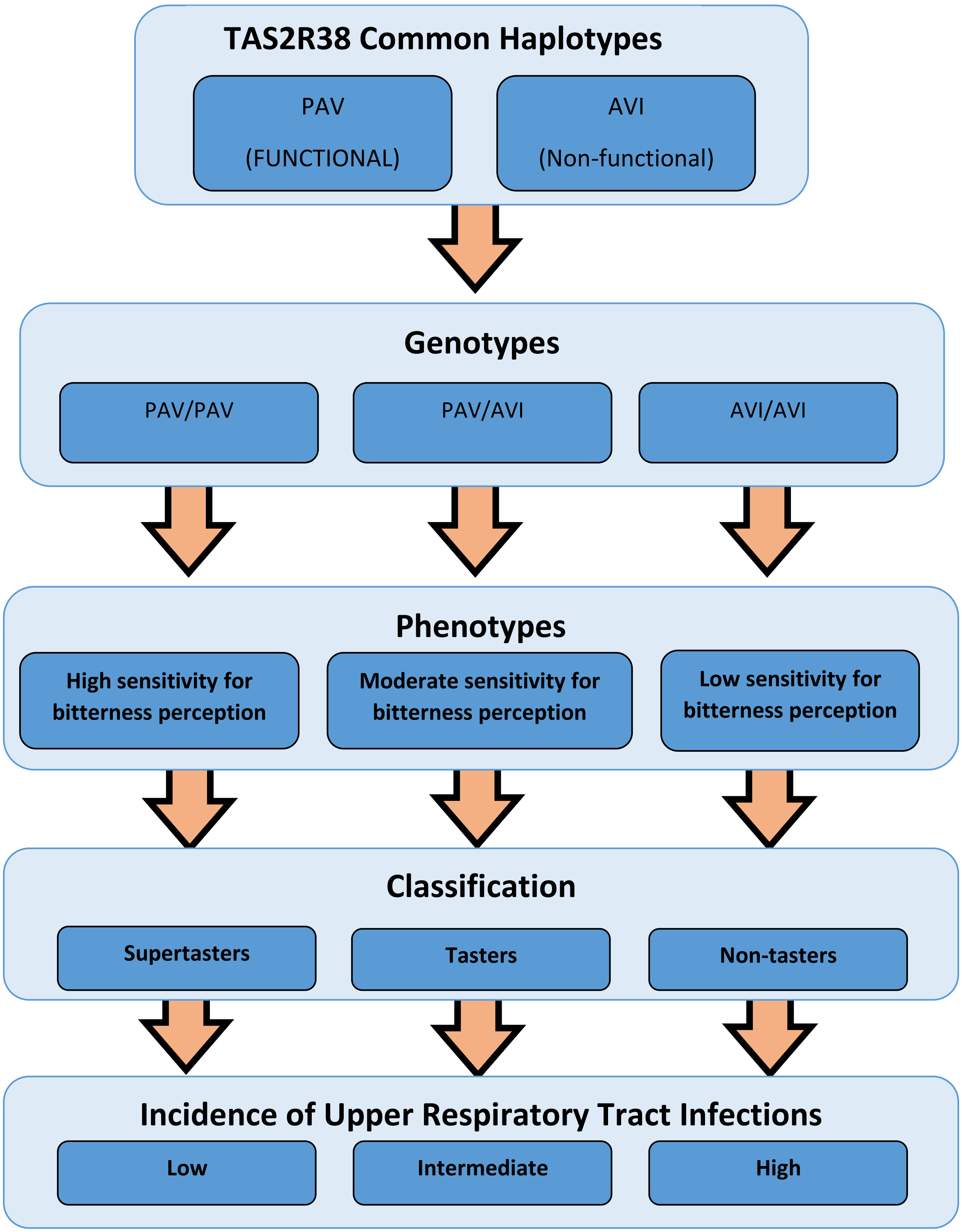

eFigure 1. Schematic Diagram Showing How Classification Into the Different Taste Groups Was Conducted. TAS2R38's common halpotypes includes PAV & AVI alleles. Homozygotes for the functional allele (PAV/PAV) have high sensitivity towards the bitter taste, and so tend to avoid bitter foods; these are classified as supertasters, with higher levels of NO production and lower incidence of upper respiratory tract infections. The same concept is applied to the other two groups.

© 2021 Barham HP et al. *JAMA Network Open.*

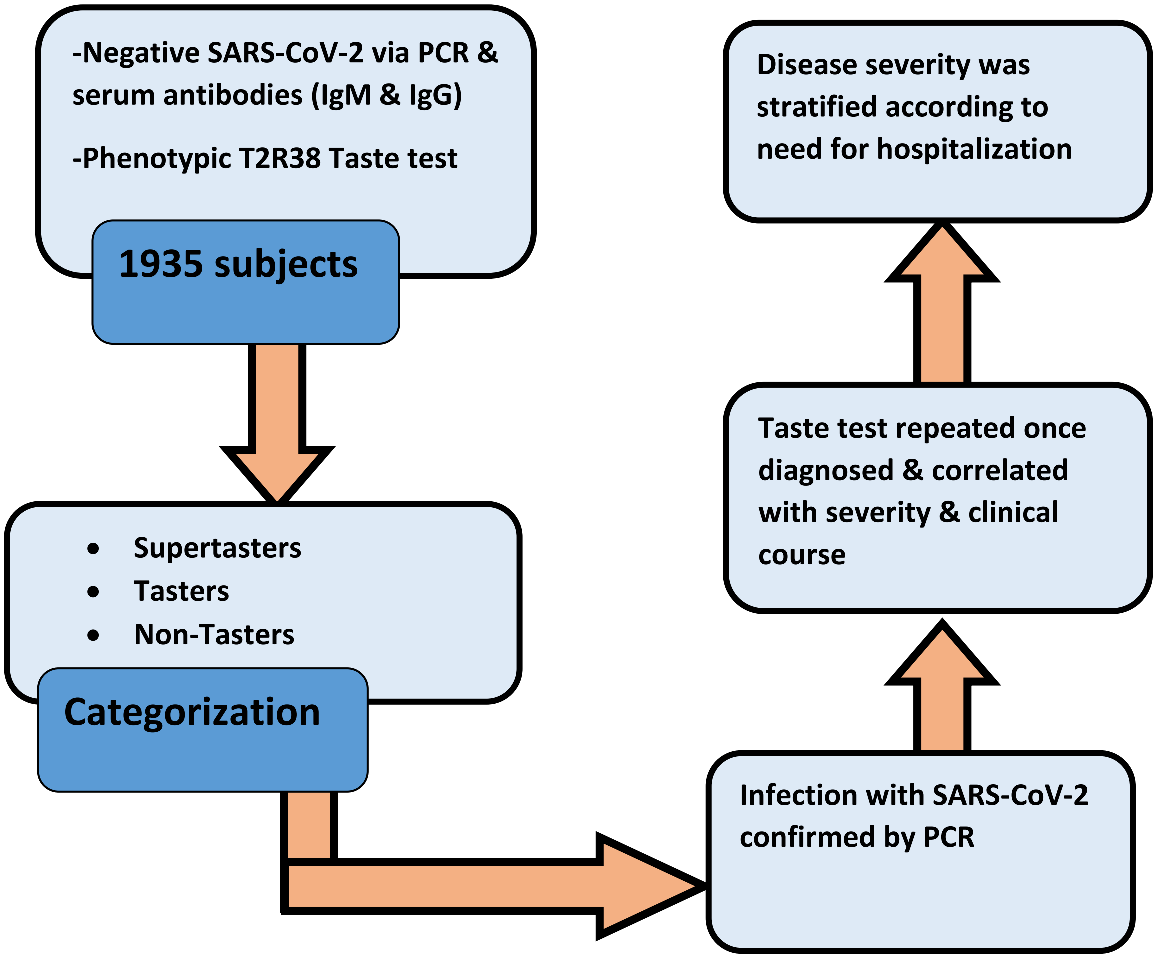

eFigure 2. Flowchart Depicting Study Design

© 2021 Barham HP et al. *JAMA Network Open.*
